# Calcium Dyshomeostasis Drives Pathophysiology and Neuronal Demise in Age-Related Neurodegenerative Diseases

**DOI:** 10.3390/ijms241713243

**Published:** 2023-08-26

**Authors:** Gerard Griffioen

**Affiliations:** ReMYND N.V., Gaston Geenslaan 1, 3001 Leuven, Belgium; gerard.griffioen@ext.remynd.com; Tel.: +32-16-75-14-26

**Keywords:** age-related neurodegeneration, calcium dyshomeostasis, Alzheimer’s disease

## Abstract

This review postulates that age-related neurodegeneration entails inappropriate activation of intrinsic pathways to enable brain plasticity through deregulated calcium (Ca^2+^) signalling. Ca^2+^ in the cytosol comprises a versatile signal controlling neuronal cell physiology to accommodate adaptive structural and functional changes of neuronal networks (neuronal plasticity) and, as such, is essential for brain function. Although disease risk factors selectively affect different neuronal cell types across age-related neurodegenerative diseases (NDDs), these appear to have in common the ability to impair the specificity of the Ca^2+^ signal. As a result, non-specific Ca^2+^ signalling facilitates the development of intraneuronal pathophysiology shared by age-related NDDs, including mitochondrial dysfunction, elevated reactive oxygen species (ROS) levels, impaired proteostasis, and decreased axonal transport, leading to even more Ca^2+^ dyshomeostasis. These core pathophysiological processes and elevated cytosolic Ca^2+^ levels comprise a self-enforcing feedforward cycle inevitably spiralling toward high levels of cytosolic Ca^2+^. The resultant elevated cytosolic Ca^2+^ levels ultimately gear otherwise physiological effector pathways underlying plasticity toward neuronal demise. Ageing impacts mitochondrial function indiscriminately of the neuronal cell type and, therefore, contributes to the feedforward cycle of pathophysiology development seen in all age-related NDDs. From this perspective, therapeutic interventions to safely restore Ca^2+^ homeostasis would mitigate the excessive activation of neuronal destruction pathways and, therefore, are expected to have promising neuroprotective potential.

## 1. Introduction

Age-related neurodegenerative diseases (NDDs) have in common the dysfunction and demise of neurons in the brain, particularly in the elderly population. Depending on the type and, therefore, function of the neurons subjected to degeneration, corresponding symptoms develop. For instance, in amyotrophic lateral sclerosis (ALS), motor neurons degenerate, leading to movement abnormalities, whereas in Alzheimer’s disease (AD), neurons operating in networks for memory formation and cognition deteriorate. In some cases, the underlying cause (or at least the most prominent cause) of age-related NDDs is known, such as in familial forms of neurodegeneration. A classic example entails familial Alzheimer’s disease (fAD), in which mutations in certain risk genes result in elevated production of neurotoxic amyloid beta (Aβ). However, far more often, the cause or causes leading to neurodegeneration in individual patients are not known. Nevertheless, many risk factors have been identified that increase the likelihood of developing neurodegeneration. These risk factors entail a plethora of conditions, characteristics, or lifestyles and can be either positive or negative (protective). Typically, these risk factors have a low penetrance and, therefore, in isolation, are not sufficient to cause neurodegeneration. Rather, it appears that the accumulation of several positive risk factors and/or the absence of negative risk factors at some point overwhelm neurons’ ability to maintain cellular homeostasis for optimal function [1]. For example, whereas ageing is a prominent and universal risk factor of age-related NDDs, ageing alone is not sufficient to cause neurodegeneration, as many people age without developing neurodegeneration. However, in combination with other low-penetrant risk factors such as, hypothetically, a sedentary lifestyle, smoking, and traumatic brain injury, it may further increase the risk to a level that neurodegeneration ensues, leading to AD symptoms during the lifetime of the individual involved.

A salient and poorly understood topic in the field is that, although typically all neurons of the CNS are exposed to, experience, or have experienced the same risk factors, typically, a selective population of neurons is the most vulnerable; hence, the different symptoms across NDDs. For instance, in the case of the fAD gene mutations mentioned above, hippocampal and cortical neurons are among the most vulnerable neurons, even though, obviously, all neurons carry these mutations. This is similar to ALS, in which specific risk gene mutations selectively affect motor neurons. Another notable observation is that although NDD risk factors are very diverse in nature and can impact different neuronal cell types, the intraneuronal pathophysiology in the affected neurons is remarkably similar, and neurodegeneration is the common outcome.

Hence, important conceptual questions about the mechanisms of neurodegeneration are unanswered or poorly understood. What molecular mechanisms enable or execute neurodegeneration? What are the common elements of such mechanisms across different neurodegenerative diseases? What is the role of ageing? What underlies selective neuronal vulnerability? Addressing these questions will increase our understanding of mechanisms underlying age-related neurodegeneration and possibly facilitate drug discovery and development approaches to counter neurodegeneration in patients. This brief review aims to provide new perspectives in a rather conceptual manner to progress our thinking about these important questions.

## 2. Making and Breaking Neuronal Connections: An Intrinsic Property of the Brain

Neurodegeneration is not a passive phenomenon that occurs spontaneously under certain pathological or adverse conditions; it entails active processes and pathways geared towards the elimination of neurons and/or neuronal structures (see Table 1 with references). This concept highlights a fundamental intrinsic characteristic of the brain: remodelling and reshaping brain networks to establish new connections while eliminating dysfunctional or underused connections. This process, known as brain plasticity [2,3], allows the nervous system to change and adapt in response to intrinsic or extrinsic stimuli and, as such, is key for brain function. For instance, the formation of new memories entails a structural change in which new synaptic connections are made or strengthened. On the other hand, during sleep, pruning of unused or dysfunctional synapses makes free space for new synaptic connections. An extreme example of brain plasticity is the developing brain in which many new connections are established but at the same time features massive destruction of about 50% of the postmitotic neurons [4].

Considering the brain operates through intrinsic physiological processes which both “make or break” neuronal structures implies a careful balance of these processes to secure optimal brain plasticity and, hence, brain function. From this perspective, when assuming pathological or adverse conditions triggering an imbalance of these intrinsic processes toward excessive destruction of neurons and/or neuronal structures, neurodegeneration would be the expected outcome. In other words, the context in which these processes operate specifies whether the outcome is beneficial or detrimental. For instance, the developing brain has features reminiscent of an AD brain, such as tau hyperphosphorylation and even tau aggregation [58], thus appearing to reflect common mechanisms underlying physiological brain plasticity during development and neurodegeneration in disease. Similarly, during hibernation, a physiological context of extensive brain remodelling, tau phosphorylation, a hallmark of AD, is increased [59,60]. Assuming neurodegeneration entails inappropriate regulation of intrinsic, physiological mechanisms of neurodestruction is in line with the observation that across different NDDs, the pathophysiology appears similar. A compelling example is the common pathophysiology in different NDDs, such as stroke and dementia [61,62]. Below, I will discuss in more depth the commonality of intraneuronal pathophysiology across different age-related NDDs.

Collectively, from a conceptual viewpoint, neurodegeneration may entail deregulation or imbalance of intrinsic, physiological mechanisms to enable brain plasticity. Decades of research have indicated that intracellular Ca^2+^ constitutes an important regulator of brain plasticity; therefore, it will be explored in relation to NDDs in the next section.

## 3. Intracellular Calcium: The Concentration Gradient Specifies Its Function for Better and for Worse

In neurons (like in many other cell types), cytosolic Ca^2+^ functions as an extremely versatile messenger controlling a wide variety of cellular functions and processes to ensure optimal function, including brain plasticity, as mentioned above [63]. A key feature of intracellular Ca^2+^ signalling entails the concentration gradient: relatively low Ca^2+^ levels in the cytosol and much higher concentrations in organelles such as the ER or the extracellular environment. To maintain this large and, therefore, vulnerable gradient, extensive mechanisms have evolved to transport cytosolic calcium against the concentration gradient. Physiological stimuli that alter the permeability of Ca^2+^ across membranes separating the gradient enables a transient flow of Ca^2+^ into the cytosol. In turn, this signal modulates Ca^2+^-sensitive processes or signalling pathways (effectors) to impact the physiology and function of the cell. Classic examples are receptor-mediated influx of Ca^2+^ through the inositol trisphosphate receptor from the endoplasmic reticulum (ER) or through the N-methyl-D-aspartate (NMDA) receptor at the plasma membrane upon stimulation by glutamate. Further, the kinetics of Ca^2+^ rise in the cytosol is another critical aspect of calcium control. Transient high increases in the cytosol may have different, and sometimes opposite, outcomes compared to prolonged modest elevations. For instance, whereas high transient increase potentiates synaptic strength, the opposite effect is caused by lower, more chronic elevations of cytosolic Ca^2+^ [64]. In other words, in addition to the concentration in the cytosol, the spatiotemporal properties of the calcium signal also specify the outcome [65].

The first column of Table 1 provides a selection of calcium-sensitive effectors in neurons. These effectors regulate key physiological processes to enable brain plasticity. The broad and diverse role of calcium underscores the importance of maintaining precise control of its concentration gradient. Adverse conditions that compromise this gradient result in an inappropriately specified Ca^2+^ signal, consequently leading to abnormal activity of otherwise physiological Ca^2+^ effectors, as outlined in Table 1. Many of these effectors mediate the pruning of synapses, axons, and dendrites or enable neuronal demise. In other words, in the absence of an optimal Ca^2+^ concentration gradient (i.e., Ca^2+^ dyshomeostasis), the Ca^2+^ signal in the cytosol is not properly specified, leading to non-selective and excessive activation of neuronal destruction pathways (Table 1). A key example entails the chronic activation of calcineurin, a calcium-sensitive phosphatase, which regulates the mechanism underlying synaptic depression, spine loss, and apoptosis [5,6,7].

In summary, intracellular calcium constitutes the central messenger controlling the fate and function of neurons to enable brain plasticity. However, under conditions of Ca^2+^ dyshomeostasis, the Ca^2+^ signal is not appropriately specified and geared towards neurodegeneration through excessive or incorrect activation of effector pathways.

## 4. Risk Factors of Neurodegeneration Compromise the Ca^2+^ Concentration Gradient

Maintaining the large Ca^2+^ concentration gradient is a challenging feat requiring energy (ATP), extensive calcium transport, and organellar Ca^2+^ storage systems. It is, therefore, also a fragile system sensitive to adverse conditions. CALMH1 P86L polymorphism is an example of a genetic risk factor of sAD, which, through its Ca^2+^ activity, may directly impact Ca^2+^ homeostasis in AD. Other examples include mutations in presenilin 1 and -2 [66] and the ApoE4 allele [67]. Ageing is a prominent example of non-genetic risk factors that impact Ca^2+^ homeostasis. One important consequence of ageing is the progressive decline of mitochondrial function caused by reactive oxygen species (ROS), a byproduct of electron transfer chain activity. In turn, decreased mitochondrial function limits the production of ATP required for maintaining the Ca^2+^ gradient [68,69]. Further, mitochondria take up cytosolic Ca^2+^ and act as important buffers to maintain Ca^2+^ homeostasis. Under conditions of excessive levels of calcium in the cytosol, the consequential excessive accumulation of intra-mitochondrial Ca^2+^ levels facilitates elevated ROS production, hence further contributing to ROS levels in the cell [70,71]. Further, elevated ROS oxidises and thereby lowers Ca^2+^ transporter activities necessary to maintain the Ca^2+^ gradient [43]. At some point, the Ca^2+^ buffering capacity of mitochondria may become overwhelmed, triggering increased opening frequency of the mPTP pore followed by a release of pro-death signals such as cytochrome C [71]. Under these conditions, mitochondria effectively turn into neurotoxic entities driving neuronal demise. Even more, the absence of efficient mitochondrial buffering sets the stage for even further elevations of cytosolic calcium in the cell, thus further contributing to neurodegeneration by excessive activation of Ca^2+^-sensitive destruction pathways, as listed in Table 1.

The above-outlined scenario illustrates a feedforward loop in that mitochondrial dysfunction, ROS production, and Ca^2+^ dyshomeostasis are intertwined, reinforcing processes [44,71], which, once out of control, spiral to abnormally high, potentially even catastrophic levels of cytosolic calcium. Such a sequence of events is well illustrated after acute brain trauma. The primary physical insult causes a dramatic release of glutamate from damaged neurons, a neurotransmitter that activates glutamatergic receptors, leading to an accordingly massive influx of calcium in the cytosol of neighbouring neurons, ultimately leading to neuronal demise (known as excitotoxicity) mechanistically similar to that explained above [72]. This, in turn, may lead to even further release of glutamate (sometimes referred to as “glutamate storm”), propagating the destruction of neurons beyond the primary lesion [73].

In the case of AD, the most prominent age-related NDD, a central role of Ca^2+^ dyshomeostasis has been proposed [1,34,64,74,75,76]. Risk factors of AD, which, unlike in the case of acute brain trauma, are wildly divergent, appear to have in common the impact, in one way or the other, of the Ca^2+^ concentration gradient [1,77]. Likewise, the risk gene mutations in fAD leading to elevated formation of neurotoxic Aβ assemblies disrupt the calcium concentration gradient to enable neurodegeneration [78,79,80]. Also, the neurotoxicity of pathological tau, another key hallmark of AD, involves calcium dyshomeostasis, at least in part through Ca^2+^ influx by potentiating NMDA receptor activity [81,82]. In AD, an even more central role of abnormally elevated cytosolic Ca^2+^ in neurodegeneration is illustrated by the observations that impaired calcium homeostasis in itself facilitates the formation of tau and Aβ pathology [75,78,83]. In other words, calcium dyshomeostasis is the cause and consequence of Aβ/tau pathology and further contributes to the development of intraneuronal pathophysiologies, such as mitochondrial dysfunction and elevated ROS production, in a reciprocal fashion, as outlined above [1].

**Figure 1:**
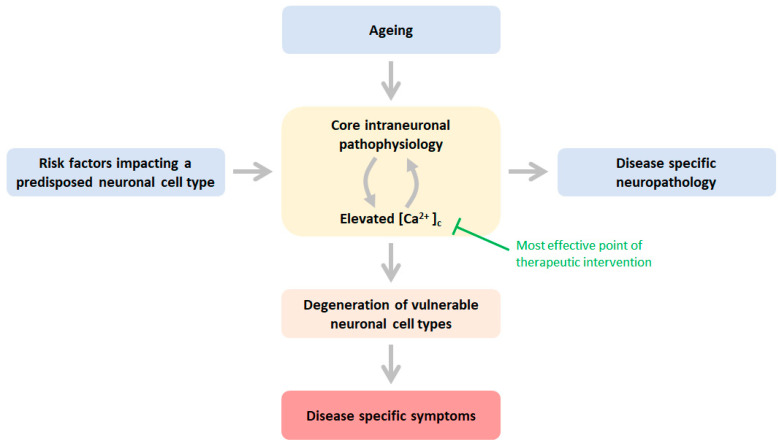
Schematic of the mechanisms underlying neuronal cell-type specific age-related neurodegeneration NDD risk factors impact directly or indirectly one or more core physiological processes in vulnerable neuronal populations predisposed to these risk factors: mitochondrial dysfunction, elevated ROS production, impaired proteostasis, decreased axonal trafficking, and calcium dyshomeostasis. Ageing is a risk factor that affects the pathophysiology indiscriminately of neuronal identity. These pathophysiological processes are engaged with cytosolic calcium in a vicious feedforward cycle, ultimately leading to excessive activation of Ca^2+^ effector pathways (Table 1). This cascade of events enables destruction and dysfunction of the vulnerable neuronal cell type, and consequently, associated neuronal cell type-specific neuropathology develops, leading to disease-specific symptoms. Green text indicates the most effective point of a therapeutic intervention entailing mitigation or prevention of an abnormal rise in Ca^2+^ in the cytosol. See text for more details.

In a scenario in which pathophysiology and elevated calcium dyshomeostasis bidirectionally reinforce each other, it follows that disease and/or patient-specific risk factors ultimately converge to Ca^2+^ dyshomeostasis and, consequently, neurodegeneration (Figure 1) through excessive activation of neuronal regression pathways (Table 1). This has led to the proposition that, at least in the case of AD, risk factors are risk factors because they impair or challenge the mechanisms underlying Ca^2+^ homeostasis [1]. This notion predicts that the penetrance of risk factors is determined by the extent to which risk factors impact Ca^2+^ homeostasis. For instance, highly penetrant and early-onset-causing risk factors, such as elevated formation of neurotoxic Aβ, are expected to have a more aggressive impact on Ca^2+^ homeostasis than risk factors with low penetrance, such as ageing. Given the central role of Ca^2+^ in controlling function and survival, it comes as no surprise that in NDDs other than AD, Ca^2+^ dysregulation drives the formation of the pathophysiology and neurodegeneration [44,45,84,85,86] (further discussed below). Examples of this include spinocerebellar ataxia [87], frontotemporal dementia [88], or Creutzfeldt–Jakob disease [89].

In summary, the risk factors of NDDs appear to have in common the ability to set off a vicious cycle in which calcium dyshomeostasis drives the pathophysiology and vice versa, ultimately spiralling to suboptimal or even catastrophic levels of cytosolic Ca^2+^ geared toward excessive activation of neuronal destruction pathways and processes (Figure 1).

## 5. Selective Neuronal Vulnerability to Risk Factors (Except Ageing)

One of the least understood questions in the field relates to the selective vulnerability of certain neuronal cell types to disease-causing risk factors, whereas typically, these risk factors are not neuronal cell type-specific. For instance, clinical mutations in early-onset genes will be present in every neuron, yet their effects often impact certain neuronal subpopulations. Alternatively, persistently elevated glucocorticoids, a risk factor of AD caused by chronic stress [90], are likely exposing all neurons, yet hippocampal neurons are among the most vulnerable neurons. Although the answers to these questions are largely elusive, one can envisage, conceptually at least, two general conditions or prerequisites underlying the selective vulnerability. The first one may entail an inherent specific attribute of neurons that causes them to be impacted more strongly than other neuronal cell types by the respective risk factor (“intrinsic predisposition”). For instance, in the example above regarding stress hormones, neurons with a relatively high density of functional stress hormone receptors at the plasma membrane [90] are expected to be more strongly impacted by glucocorticoids than neurons that have low or no glucocorticoid signalling; PD risk factor MPTP targets selectively dopaminergic neurons because dopamine transporter (DAT), which transports MPTP across the plasma membrane [91], is exclusively present in this type of neuron.

A second condition one can envisage is to what extent risk factors impact the most limited process for a specific neuron type for optimal function (“physiological predisposition”). In other words, what is the weakest link affected by adverse conditions? For instance, specific cellular or physiological determinants attributed to the neuronal identity confer selective vulnerability to alpha-synuclein or tau toxicity [92]. In the MPTP example above, the weakest link in dopaminergic neurons might be the antioxidant defence. This function is relatively stretched, given that dopaminergic neurons have a high oxidative stress load because of high ROS production associated with dopamine metabolism [93], and further because the levels of Fe^2+/3+^, a redox-active metal ion is accumulated in this type of neuron [94,95]. If, then, MPTP, a mitochondrial toxin, triggers excessive mitochondrial ROS production, this might be the “last straw that breaks the camel’s back”, overwhelming the antioxidant defence and triggering neuronal demise through the mechanisms explained above. Likewise, in ALS, the risk factors affecting axonal transport or processes requiring axonal transport are possibly the most sensitive to motor neurons because of their very long axons, which imposes huge challenges for axonal transporting systems to maintain functional axonal terminals [96,97]. Alternatively, in the case of AD, vulnerable neurons in the hippocampus are relatively sensitive to hypoxia [8], possibly because this neuronal type appears to consume oxygen at a relatively high rate, given that the hippocampus is amongst the most vascularised brain regions [98].

Irrespective of the above, ageing is a universal risk factor for age-related neurodegenerative disorders. In other words, it seems likely that ageing impacts pathophysiological elements shared by age-related NDDs. As outlined above, mitochondrial dysfunction is a prominent mechanism of biological ageing, suggesting the pathophysiology directly or indirectly associated with it (such as oxidative stress, Ca^2+^ dyshomeostasis, ER stress) underlies the common intraneuronal pathophysiology across NDDs.

Thus, intrinsic and physiological predispositions to NDD risk factors may comprise at least two characteristics underlying neuronal selectivity. Although such anticipated predispositions are, at this point, rather hypothetical, they may provide a conceptual context to understand the selective vulnerability of neurons. For instance, what intrinsic and/or functional dispositions may medium spiny neurons have to account for the selectivity towards mutant huntingtin in HD? Or motor neurons carrying SOD1 clinical mutations for ALS? Ageing is a universal risk factor (for age-related NDDs), indicating that mitochondrial dysfunction and associated pathophysiology comprise common mechanisms underlying age-related neurodegeneration.

## 6. Different Risk Factors Impacting Different Neuronal Cell Types but Yet a Common Outcome

Although disease risk factors and the correspondingly affected neuronal cell types differ across neurodegenerative diseases, surprisingly, the intraneuronal pathophysiology in the diseased neurons is remarkably similar. Prominent common “core” elements of intraneuronal pathophysiologies entail mitochondrial dysfunction, increased oxidative stress, calcium dyshomeostasis, reduced axonal trafficking, and impaired proteostasis [44,99,100,101].

As discussed above, at least a subset of these core pathophysiological processes are engaged with Ca^2+^ dyshomeostasis in a vicious feedforward cycle. Mitochondrial dysfunction with associated ROS production, impaired proteostasis, or calcium-induced calcium release (CICR) are prominent examples in which the cause and consequence of Ca^2+^ is dyshomeostasis. This is not surprising given the central role of Ca^2+^ in virtually all aspects of cell physiology, including those that control Ca^2+^ homeostasis. However, although the pathophysiology has common core elements, it appears that the neuropathology is (at least to some extent) disease-specific. For instance, the formation of Aβ pathology in cortical neurons in AD does not seem to occur in motor neurons in the case of ALS. Alternatively, aggregation of huntingtin is only observed in HD. Apparently, the specific outcome of shared pathophysiological processes, such as impaired or limited proteostasis, can result in a neuronal cell type and, thus, disease-specific protein deposition pathology (Figure 1).

Collectively, risk factors of neurodegeneration may promote one or more elements of the shared pathophysiology (Figure 1). Given the interdependent and reinforcing nature of these core intraneuronal pathophysiological processes (mitochondrial dysfunction, ROS production, impaired proteostasis, reduced axonal transport, and Ca^2+^ dyshomeostasis), it ultimately leads to similar or overlapping pathophysiology across neurodegenerative diseases. An inevitable and common outcome constitutes a suboptimal or even a catastrophic rise of cytosolic calcium, executing neurodegeneration through the excessive activation of intrinsic mechanisms of neuronal demise (Table 1). Ageing, through an impact on mitochondrial health, constitutes a risk factor impacting a core pathophysiological process in a neuronal cell-type indiscriminate manner and, as such, constitutes a risk factor shared by all age-related neurodegenerative diseases.

## 7. Therapeutic Approaches: Restoring the Distorted Calcium Gradient

The mechanism outlined in Figure 1 illustrates that risk factors of neurodegenerative diseases trigger pathophysiology in a feedforward fashion in conjunction with cytosolic Ca^2+,^ leading to neurodegeneration. From this follows that therapeutic interventions that mitigate or prevent the pathological rise of cytosolic Ca^2+^ are expected to have a large therapeutic potential across different NDDs. After all, such an approach would break most effectively the anticipated vicious cycles of core pathophysiology processes leading to neuronal death. In other words, such interventions would enable neurons to be more resilient to adverse disease risk factors.

From a more practical perspective, however, approaches to normalising Ca^2+^ homeostasis safely are challenging precisely because Ca^2+^ has such a central role in regulating physiology, not only in neurons but in virtually all cells of the body. Targeting specific modulators of Ca^2+^ homeostasis (Table 2), such as, for example, specific calcium transporters or ionotropic receptors, typically has two major shortcomings. Firstly, their inhibition may also impact their physiological function and, thus, could lead to undesirable side effects unless it is done in a controlled, partial manner. Secondly, targeting only one specific player controlling Ca^2+^ homeostasis may be too limited to achieve a clinically meaningful lowering effect on cytosolic calcium because calcium dyshomeostasis is controlled by a multitude of different processes. An excellent example illustrating these points is memantine, one of the therapeutic options in AD (Table 2). This drug partially antagonises the NMDA receptor, as full inhibition most likely would lead to unwanted side effects, and the NMDAR is just one of the many players leading to Ca^2+^ dyshomeostasis in AD. Hence, although conceptually it illustrates that restoring Ca^2+^ homeostasis is a valid approach to treating AD, at the same time, these limitations seem to limit the effect size in patients.

At present, no clinically validated therapeutic concepts are known that effectively normalise calcium homeostasis in diseased neurons of patients in a safe way. Some concepts or candidate drugs under study are listed in Table 2. However, one approach that comes close entails chronic treatment with calcineurin inhibitors cyclosporine and FK506. Calcineurin (CN) is a prominent Ca^2+^-sensitive phosphatase that extensively regulates neuronal functionality and survival. In animal models of AD, CN inhibition restores synaptic plasticity with a large effect size [9,120]. In a retrospective analysis, chronic administration of calcineurin inhibitors cyclosporine and tacrolimus to patients who had undergone organ transplantation were virtually fully spared from developing dementia compared to the general population [10]. These data suggest that normalising calcium homeostasis is a promising approach for treating neurodegenerative diseases, especially because this would not only normalise CN activity but all deranged calcium-sensitive effector pathways mediating neurodegeneration (Table 1). Unfortunately, the systemic side effects of direct inhibition of CN are quite severe and may preclude using CN inhibitors as drugs for treating NNDs unless perhaps patient-compliant CNS delivery methods, such as nasal or intrathecal administration, become available.

## 8. Conclusions

Collectively, this review outlines a mechanism of neurodegeneration in which risk factors of neurodegeneration impact directly or indirectly one or more core physiological processes in vulnerable neuronal populations predisposed to these risk factors: mitochondrial dysfunction, elevated ROS production, impaired proteostasis, decreased axonal trafficking, and calcium dyshomeostasis. Ageing is a risk factor that contributes to thepathophysiology indiscriminately of neuronal cell type. These pathophysiological processes are engaged with cytosolic calcium in a vicious feedforward cycle, which ultimately leads to excessive activation of Ca^2+^ effector pathways geared toward neuronal destruction of vulnerable neurons. Consequently, normalising Ca^2+^ homeostasis would be the most effective therapeutic intervention, provided it does not overly impact physiological calcium signalling.

## Figures and Tables

**Table 1:** The anticipated impact of Ca^2+^ dyshomeostasis on neuronal structures, function, and survival. Effectors are listed alphabetically.

Ca^2+^ Sensitive Effectors (Not Exhaustive)	Anticipated Intraneuronal Pathophysiology/Neuronal Destruction Pathways under Conditions of Ca^2+^ Dyshomeostasis	References
**Calcineurin**	Synaptic depression, dendritic spine loss, apoptosis, altered mitochondrial dynamics, inhibition of axonal outgrowth	[5,6,7,8,9,10,11,12,13,14,15,16,17,18,19,20]
**Calpains**	Dendritic pruning, axonal degeneration	[21,22,23,24,25]
**Calpain-p25-CDK5** **Calpain-GSK3** **Calpain-tau**	Apoptosis, phosphorylation tau, neurotoxic Aβ generation, synaptic dysfunction, mitochondrial dysfunction, excessive ROS production, neurotoxic tau fragments	[26,27,28,29]
**CamKII**	Phosphorylation tau, apoptosis, necrosis, synaptic degeneration	[30,31]
**ER function**	Impaired proteostasis, ER-stress-induced apoptosis (because of depleted Ca^2+^ levels in the ER)	[32,33]
**IP_3_ and ryanodine receptors**	Ca^2+^ dyshomeostasis (by elevated CICR)	[34,35,36,37]
**MAPK (ERK)**	Apoptosis	[38,39,40,41]
**Miro**	Stalled axonal trafficking mitochondria	[42]
**Mitochondrial function**	Ca^2+^ dyshomeostasis, apoptosis, necroptosis, excessive ROS production, ATP production	[43,44,44,45,46,47]
**Proteasome**	Axonal degeneration	[48,49]
**RIPK**	Necroptosis	[22,50]
**vATPase function**	Impaired lysosomal-autophagosome function	[51]
**Vesicular trafficking**	APP processing, tau-exocytosis	[52,53,54,55,56,57]

**Table 2:** Small molecule modulators of calcium homeostasis and their effects. Compounds are listed alphabetically.

Compound	Target	Effects in Non-Clinical Models and (Where Indicated) in Patients	References
Dantrolene	RyR	Reduces amyloid pathology, normalises synaptic plasticity, and improves behavioural performance.	[102,103,104]
Isradipine, nimodipine, nitrendipine	Ca_v_1.2 channel	Reduces amyloid and tau pathology, improves autophagy, and mitigates cognitive impairment. Possibly some benefit in patients.	[105,106,107,108,109]
Levetiracetam	SV2a	Mitigates network hyperactivity and improves learning and memory.	[110]
Memantine	NMDA receptor	Dendritic spine regeneration, rescue of synaptic plasticity, reduced hippocampal CA1 neuron loss reduction Aβ/tau pathology, and improved learning and memory performance. Benefits cognitive, functional, global, and behavioural endpoints in patients.	[111,112,113,114]
NDC-1173, CDN1163	SERCA pump activator	Improves memory and other behavioural read-outs.	[115,116]
REM0046127	SOCE modulator	Full rescue of synaptic plasticity, EEG, and cognition. Reduces inflammation and Aβ/tau pathology.	Personal communication GG
S107 (Rycal)	RyR_2_ macromolecular complex	Reduces APP cleavage and Aβ production and restores synaptic plasticity and cognitive deficits.	[117,118]
TG-2112x	Lowers mitochondrial Ca^2+^ uptake	Mitigates glutamate excitotoxicity.	[119]

Abbreviations: RyR: ryanodine receptors; SV2a: synaptic vesicle glycoprotein 2A; NMDA: N-methyl-D-aspartate; SERCA: Sarcoendoplasmic reticulum calcium ATPase.

## Data Availability

Not applicable.
